# Predicting Survival After Bone Marrow Transplant Using Time Series of Routine Laboratory Biomarkers

**DOI:** 10.1093/geroni/igaa057.2715

**Published:** 2020-12-16

**Authors:** Ravi Varadhan, Bohao Tang, Hua-Ling Tsai, Phil Imus

**Affiliations:** Johns Hopkins University, Baltimore, Maryland, United States

## Abstract

Bone marrow transplant (BMT) is a curative therapy for patients with hematologic malignancies. However, there is still a high rate of relapse and mortality after BMT. It would be tremendously valuable if we can identify older adults at high-risk for mortality using readily available information. A number of biomarkers are routinely collected during follow-up for clinical care, but this information is seldom used in prediction models. We examined the data from 1011 patients who had BMT at Johns Hopkins between 2013 and 2019. There were 364 death over a median follow-up of 431 days. We considered 4 biomarkers: albumin, hemoglobin, lymphocytes count, and platelets. Biomarker data from one week pre-BMT to 8 weeks post-BMT was used for prediction using a random survival forest model. The model performed quite well and had a 5-fold cross-validated c-index of 0.733 (95%CI: 0.724-0.739). Routine laboratory biomarkers can help identify poorly resilient older BMT patients.

